# African wild dogs (*Lycaon pictus*) from the Kruger National Park, South Africa are currently not inbred but have low genomic diversity

**DOI:** 10.1038/s41598-022-19025-7

**Published:** 2022-09-02

**Authors:** Christina Meiring, Haiko Schurz, Paul van Helden, Eileen Hoal, Gerard Tromp, Craig Kinnear, Léanie Kleynhans, Brigitte Glanzmann, Louis van Schalkwyk, Michele Miller, Marlo Möller

**Affiliations:** 1 DSI-NRF Centre of Excellence for Biomedical Tuberculosis Research, South African Medical Research Council Centre for Tuberculosis Research, Division of Molecular Biology and Human Genetics, Faculty of Medicine and Health Sciences, Stellenbosch University, PO Box 241, Francie van Zijl Drive, Cape Town, 7500 South Africa; 2 South African Tuberculosis Bioinformatics Initiative (SATBBI), Faculty of Medicine and Health Sciences, Stellenbosch University, Francie van Zijl Drive, PO Box 241, Cape Town, 7500 South Africa; 3 Genomics Centre, South African Medical Research Council, Francie van Zijl Drive, PO Box 19070, Cape Town, 7500 South Africa; 4 Department of Agriculture, Land Reform and Rural Development, PO Box 12, Skukuza, 1350 South Africa; 5 Department of Veterinary Tropical Diseases, Faculty of Veterinary Science, University of Pretoria, Private Bag X04, Soutpan Road, Pretoria, 0110 South Africa; 6 Department of Migration, Max Planck Institute of Animal Behavior, Am Obstberg 1, 78315 Radolfzell, Germany; 7 Centre for Bioinformatics and Computational Biology, Stellenbosch University, Private bag X1, Merriman Avenue, Stellenbosch, 7600 South Africa

**Keywords:** Computational biology and bioinformatics, Genetics

## Abstract

African wild dogs (*Lycaon pictus*) have undergone severe population reductions and are listed as endangered on the International Union for Conservation of Nature Red List. Small, isolated populations have the potential to suffer from threats to their genetic diversity that may impact species viability and future survival. This study provides the first set of population-wide genomic data to address conservation concerns for this endangered species. Whole genome sequencing data were generated for 71 free-ranging African wild dogs from the Kruger National Park (KNP), South Africa, and used to estimate important population genomic parameters. Genomic diversity metrics revealed that variation levels were low; however, this African wild dog population showed low levels of inbreeding. Very few first- and second-order relationships were observed in this cohort, with most relationships falling into the third-order or distant category. Patterns of homozygosity could have resulted from historical inbreeding or a loss in genome variation due to a population bottleneck. Although the results suggest that this stronghold African wild dog population maintains low levels of inbreeding, likely due to their cooperative breeding system, it may lead to a continuous population decline when a reduced number of suitable mates are available. Consequently, the low genomic variation may influence species viability over time. This study highlights the importance of assessing population genomic parameters to set conservation priorities. Future studies should include the investigation of the potential of this endangered species to adapt to environmental changes considering the low genomic diversity in this population.

## Introduction

The global African wild dog (*Lycaon pictus*) population has declined significantly in number and geographical distribution; it is currently found in only 14 of the 39 countries where it historically occurred^1–4^. The major factors driving the decline of African wild dogs are human-wildlife conflict (persecution and snaring), interspecies conflict (predation by lions and hyenas), habitat loss, climate change, and infectious diseases^3,5–7^. Consequently, remnant populations are small, fragmented, and vulnerable to threats such as genetic drift, which may cause a loss of genomic variation^8–11^. This may threaten population survival since low genetic variation can impact reproductive success, increase susceptibility to disease, and limit the ability to respond to environmental changes^12,13^.

Genetic studies conducted on African wild dogs in the context of conservation management have used markers such as microsatellites and mitochondrial DNA or targeted gene regions to investigate genetic variation^10,14–19^. Most of these investigations reported low levels of genetic diversity across different populations, including African wild dogs from the Kruger National Park (KNP), which is the largest population in South Africa^14,19,20^. Inbreeding in African wild dogs has also been studied, since mating between relatives may occur when the number of unrelated mates in the remaining populations is low^17^. Spiering et al. (2012) confirmed the occurrence of inbreeding in a population from the KwaZulu-Natal (KZN) province in South Africa, but only in a limited number of packs. Even though no evidence of inbreeding depression, such as reduced fitness or impacts on reproductive success, was identified, they found that African wild dogs with a certain level of inbreeding (*F* ≥ 0.25), had reduced lifespans^17^. Another study indicated that inbreeding avoidance occurred in a reintroduced African wild dog population in KZN and highlighted this as an important factor to consider when planning conservation strategies^18^.

Recently, studies have utilised genomic data to address important questions regarding the genetic diversity, population history, adaptations, and demographic history of African wild dog populations^21–23^. These investigations allowed for the construction of an African wild dog reference genome^21^, the ability to conduct genome-wide scans to identify segments of genome responsible for adaptation, and to improve accuracy of important conservation genomic parameters in African wild dog populations. However, assessments of genomic variation, and estimations of inbreeding and relatedness (using WGS data) have not been conducted on a population-level. The generation of this information may provide the opportunity for effective actions if integrated into conservation planning.

Whole genome sequencing (WGS) has allowed the use of genome-wide markers to accurately assess the genomic diversity and other population genetic parameters in conservation research^24–27^. Single nucleotide polymorphisms (SNPs) are markers that represent the sequence variation of an individual at a genome-wide scale and have proven useful in several conservation genomic studies^28–32^. Specifically, SNPs have been used to accurately estimate population-level diversity, gain insight into the demographic history of a population, and provide valuable information regarding population sizes, mating systems, relatedness, population structure, and dispersal rates^33,34^. These parameters are important when planning conservation interventions for wildlife such as population management and monitoring programs^35^.

Other parameters, such as levels of inbreeding that result from mating between related individuals are also important to investigate, since high levels can lead to a reduction in fitness, which may impact population growth and viability^29,36^. Estimates of relatedness among African wild dogs, based on identity-by-descent (IBD), can potentially be determined with whole genome SNP data in the absence of pedigree data^27^. The quantification of inbreeding and relatedness has implications for African wild dog conservation efforts and should be evaluated to ensure positive population growth and maintenance of genomic diversity^37,38^. The identification of homozygous segments in the genome (resulting from inbreeding) is also facilitated by WGS and can be used to distinguish between recent and distant inbreeding^6,39^. Furthermore, the inbreeding coefficient of a population can be calculated using genome-wide SNPs, based on runs of homozygosity (ROH) detection (F_ROH_) and the canonical estimate based on excess SNP homozygosity (F_HOM_)^40–43^. Identifying homozygous segments and determining the inbreeding coefficient of a population is important in the field of conservation biology as it can aid in preventing inbreeding depression and therefore, improve genomic variation^26,27^.

Since an understanding of the genomic variation within a population, including relatedness and inbreeding, is essential for evaluating population viability and setting conservation priorities, this study aimed at assessing: (1) genomic diversity metrics, (2) relatedness based on IBD, and (3) inbreeding signatures (ROH) and coefficients (F_HOM_ and F_ROH_) in an African wild dog population from the KNP, South Africa. It is one of the few self-sustaining populations and therefore, it would represent the greatest likelihood of natural genomic diversity for the South African wild dog population. Additionally, establishing genome variation using WGS has not been previously undertaken at a population level in African wild dogs and will provide the foundation to develop genomic profiles which can be used for strategic population management. When considering the significant decline of African wild dog numbers from an estimated 500,000 in 1900, to 6600 today (4), we expect that any African wild dog population would have reduced levels of genome variation. Although the KNP population is the only viable population in South Africa, it is largely unmanaged^1,44^, and we expect to observe low levels of genomic diversity. Based on the sample availability for WGS, which mostly included samples from the alpha female and male of different packs, we expect to observe distant relatedness and low levels of inbreeding, since we expect that the alpha pair would mostly be unrelated. However, the occurrence of a drastic population reduction may have caused historic inbreeding signatures to remain in these African wild dog’s genomes.

## Results

### Quality control, read mapping and variant calling

Two WGS platforms based on the same sequencing principle (BGISEQ-500 and MGISEQ2000)^45^ were used to generate whole genome sequences for 71 African wild dogs from KNP at a relatively high depth of coverage (mean: 31.21$\times$, standard deviation: ± 8.10) after quality filtering and removal of duplicates^45^. The percentage bases covered at this depth was 97.45% (95% CI 96.30–98.60%) and the average percentage of reads that successfully mapped to the African wild dog reference genome^21^ was 98.82% (95% CI 97.70–100%). Variant calling was performed on all 71 genomes and 5,638,393 unique SNPs and 4,338,390 indels were identified. After quality filtering, this was reduced to 2,479,949 and 522,400 SNPs and indels, respectively. A Ts/Tv ratio of 1.79 ± 0.20 was obtained for the raw variant calls and increased to 2.02 ± 0.18 after filtering. Finally, after linkage disequilibrium (LD) pruning and filtering for minor allele frequency (MAF) and deviation from Hardy–Weinberg equilibrium (HWE), 527,726 variants remained.

### Population genomic diversity and bottleneck

**Figure 1:**
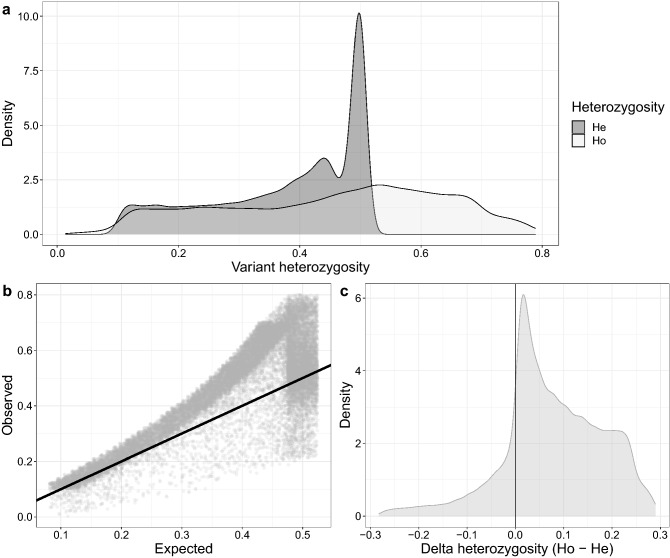
Distribution of heterozygosity across a subset of single nucleotide polymorphisms (SNPs) of whole genome sequences in African wild dogs (*Lycaon pictus)* from Kruger National Park (KNP), South Africa. (**a**) Density of observed heterozygosity (H_O_) and expected heterozygosity (H_E_) across 86,149 SNPs. (**b**) Scatter plot representing the correlation of observed and expected heterozygosity of the subset of SNPs. (**c**) Distribution of delta heterozygosity (Δ = H_O_ − H_E_) across SNPs.

The mean minor allele frequency and standard deviation of African wild dog genomes were 0.26 ± 0.13 (median: 0.25) after filtering for MAF (≤ 0.05), deviations from HWE, and LD pruning. The nucleotide diversity (π) was calculated as 0.000099 ± 0.000134 across all sites in this population. The observed heterozygosity (H_O_) was 0.39 ± 0.17 and the expected heterozygosity (H_E_) was 0.35 ± 0.12. After stringent LD pruning, 86,149 independent SNPs remained and H_O_ and H_E_ were calculated as 0.45 ± 0.18 and 0.38 ± 0.12, respectively. The distribution of the heterozygosity revealed that H_E_ conformed to that of a normal binary marker having a maximum H_E_ of 0.5; however, the H_O_ included a large proportion of values above 0.5 (Fig. 1a; Supplementary Fig. 1a). The correlation of the H_O_ and H_E_ of a subset of SNPs indicated that most markers deviated above the identity line (Fig. 1b), which is also reflected in Fig. 1c, where the density of the delta heterozygosity (Δ = H_O_ − H_E_) of independent variants was predominantly distributed above 0. Similar results were observed when the original SNP dataset was used (Supplementary Fig. 1b, c). Due to the non-normal distribution of H_O_, the excess heterozygosity statistical test (standardized difference test) was used to determine whether H_O_ was significantly larger than the expected heterozygosity. This African wild dog population demonstrated significant excess heterozygosity based on the standardized difference test which produced a T_2_ statistics equal to 142.18 (Z-score) with a significance of *P* << 0.0001 (one-sided test), indicative of a population bottleneck.

### Population structure

**Figure 2:**
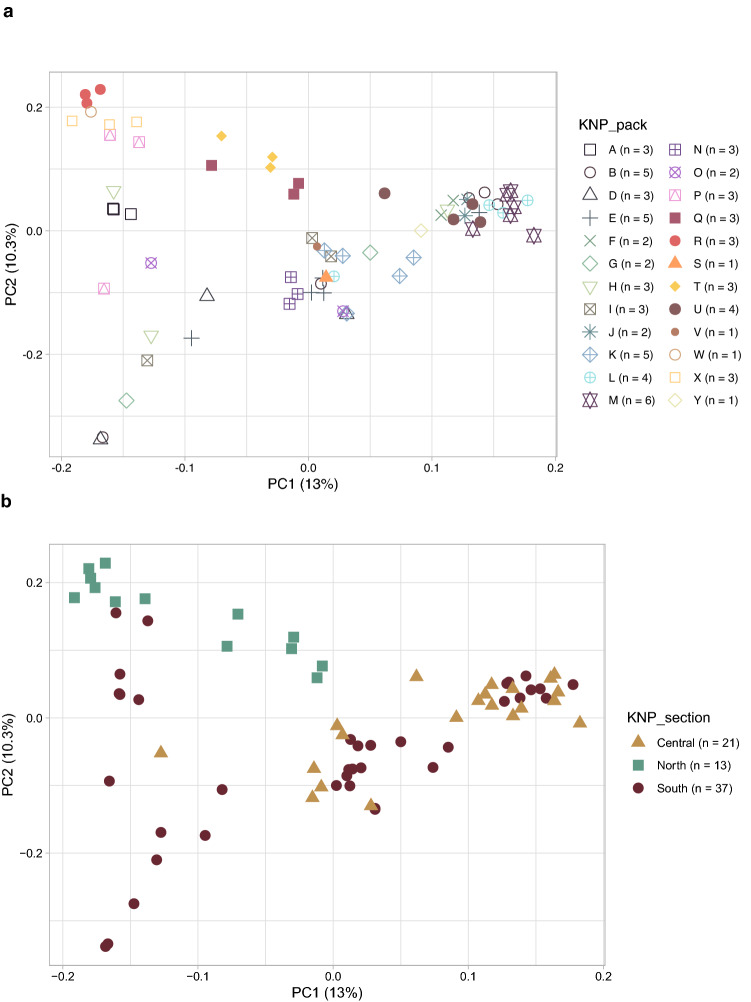
A scatterplot from a principal component analysis (PCA) of 71 African wild dog (*Lycaon pictus*) samples (24 packs) from the Kruger National Park (KNP), South Africa. The first two principal component (PC) axes 1 and 2 are shown, with the percent (%) variance accounted for by each indicated in parentheses. (**a**) Each data point in the scatterplot represents an individual, with the coloured shapes corresponding to the pack (KNP_pack). (**b**) Colours and shapes correspond to an individual’s geographical location in KNP.

To assess the genetic relationship and structure among African wild dog packs, a principal components analysis (PCA) was conducted using a SNP set (527,726 SNPs) that was filtered for MAF, LD (moderate), and deviations from HWE. The PCA was also used to investigate whether differences were introduced by the two WGS platforms. Figure 2 shows the first two principal component (PC) axes of 24 African wild dog packs, which explained 23.3% of the total variation (Supplementary Fig. 2). There was no clear separation between packs on PC axes 1 and 2 (Fig. 2a), or on PC axis 3 (Supplementary Fig. 3a and b), however, individuals from the same packs clustered closer together than individuals from different packs. When considering the geographical origins of each African wild dog within KNP, PC 2 revealed subtle separation between individuals from north, south, and central KNP with some overlap (Fig. 2b). There was no clear separation between sequencing platforms (Supplementary Fig. 4a–c).

### Relatedness

A pairwise IBD analysis was conducted to investigate the relatedness in the KNP African wild dog population. First- and second-degree, as well as more distant relationships, were explored among the wild dog individuals using PLINK v1.90^40^. The pairwise IBD analysis identified 3 African wild dog pairs out of a total of 2485 pairs (0.12%; 95% CI 0.04–0.31%) as first-degree related (parent–offspring or full sibs), none of which came from the same packs. A total of 231 pairs were classified as second-degree relatives (9.30%; 95% CI 8.21–10.50%; Table 1); however, 53 of these pairs had relatedness measures above 0.4, most of which came from the same pack. Most individuals (77.51%; 95% CI 75.82–79.10%) were found to be distantly related and 13.08% (95% CI 11.81–14.92%) of African wild dogs were completely unrelated.

**Table 1:** Summary of relationship category assignment (RCA) for African wild dogs (*Lycaon pictus*) from Kruger National Park (KNP), South Africa obtained from single nucleotide polymorphisms (SNP) allelic profiles using PLINK v1.90.

Category	Total pairs	Portion of cohort	$\overline{x }$Z_0_	$\overline{x }$Z_1_	$\overline{x }$Z_2_	$\overline{x }$PI-HAT
1st Degree relatives	3	0.12%	0.136	0.455	0.409	0.637
2nd Degree relatives	231	9.30%	0.386	0.563	0.051	0.333
Distant relatives	1926	77.51%	0.760	0.237	0.003	0.121
Unrelated	325	13.08%	1	0	0	0
Total	2485					

$\overline{x}$Z_0_, average probability to share zero identity-by-descent (IBD) alleles; $\overline{x}$Z_1_, average probability to share one IBD allele; $\overline{x}$Z_2_, average probability to share two IBD alleles; and $\overline{x}$PI-HAT, average relatedness measure.

### Inbreeding

**Figure 3:**
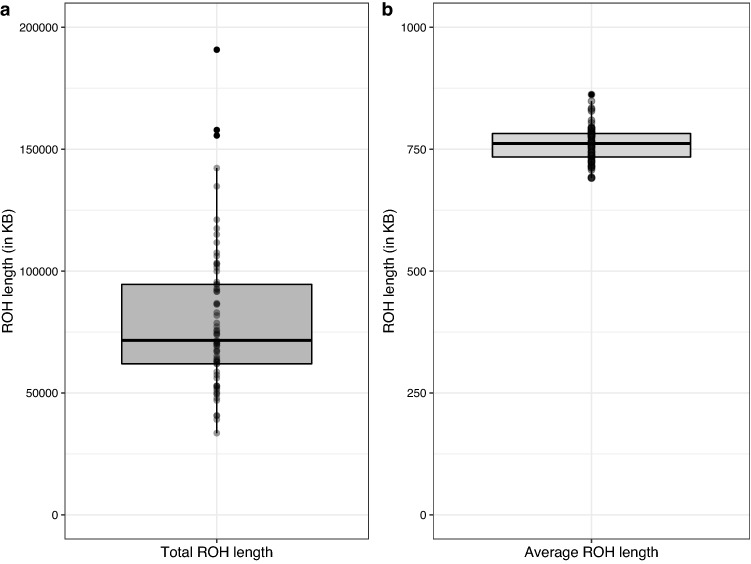
The distribution of the runs of homozygosity (ROH) lengths (kilobase pairs) of genomes in 71 African wild dogs (*Lycaon pictus*) from Kruger National Park (KNP), South Africa. (**a**) The total length of the ROH segments. (**b**) Average length of the ROH segments.

**Figure 4:**
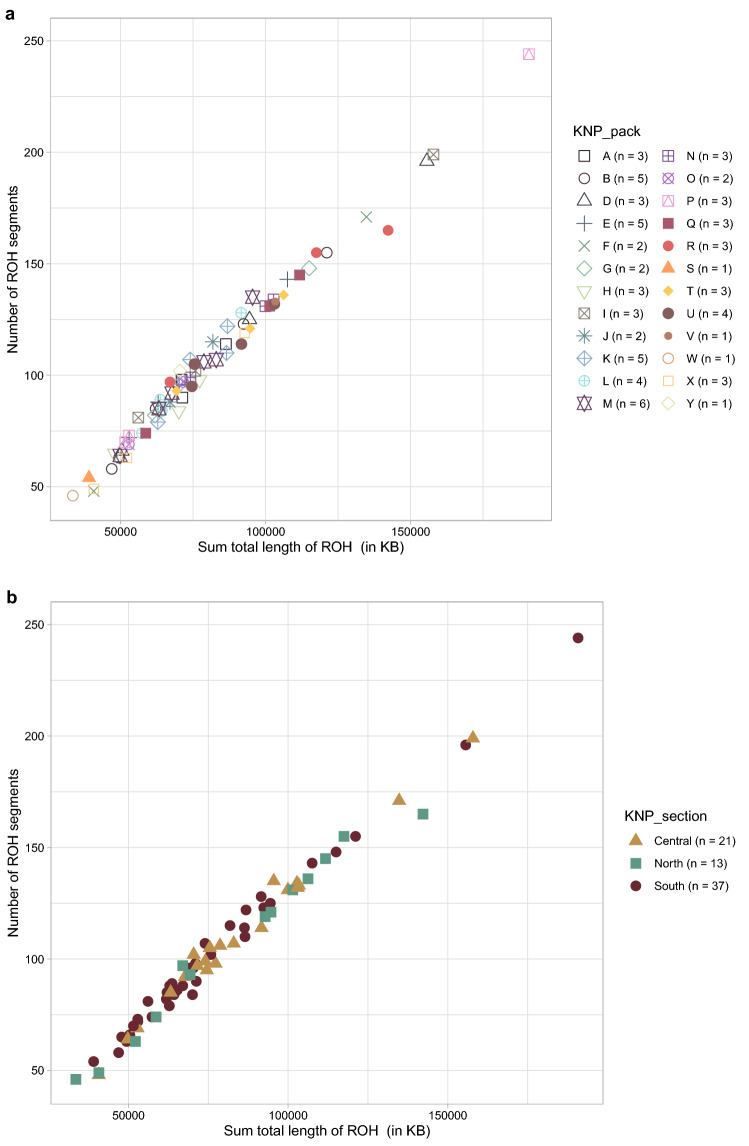
Total genomic length (kilobase pairs) covered by runs of homozygosity (ROH) per individual (x-axis) and total number of ROH per individual (y-axis) of African wild dogs (*Lycaon pictus*) from Kruger National Park (KNP), South Africa. (**a**) Each data point in the plot represents an individual, with the coloured shapes corresponding to the pack (KNP_pack). (**b**) Colours and shapes correspond to an individual’s geographical location in KNP.

**Figure 5:**
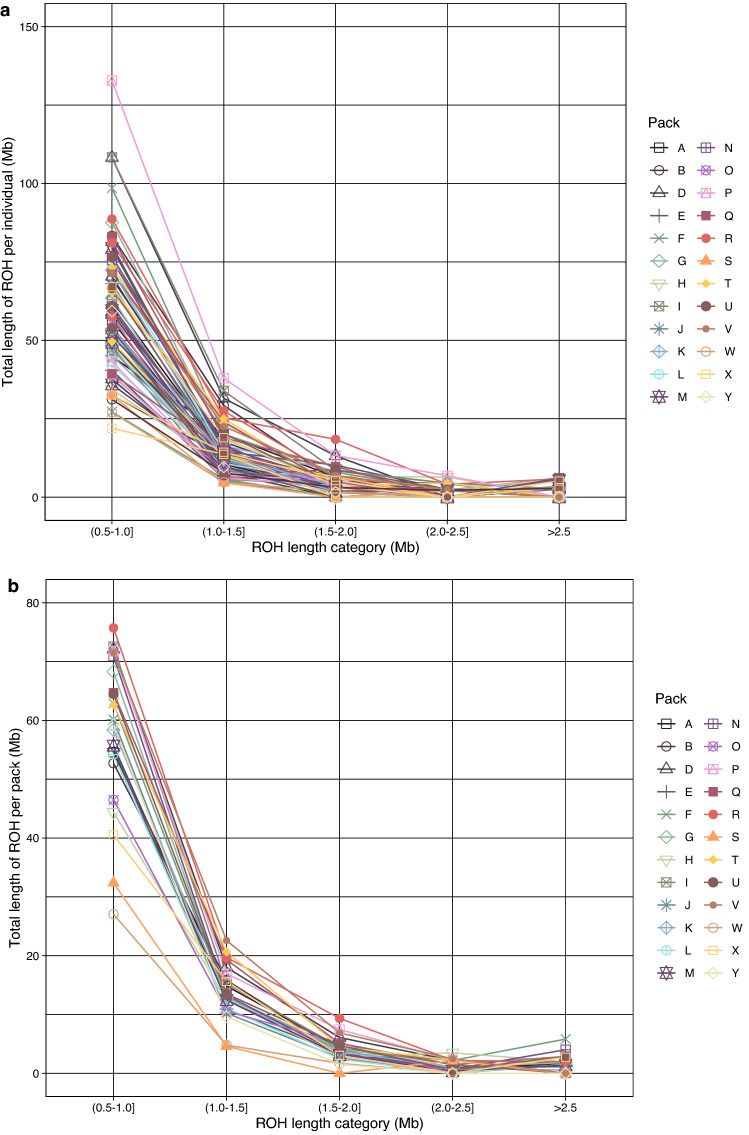
Estimated length distributions of runs of homozygosity (ROH) in 71 African wild dogs (*Lycaon pictus*) from Kruger National Park (KNP), South Africa. (**a**) The total length of ROHs mega base pairs (Mb) over five length categories, described for each African wild dog. Individuals were coloured according to pack. (**b**) The total length of ROHs (Mb) over five length categories of ROH tract lengths as in (a), calculated as the average for each pack. The colouring scheme is the same as in (**a**).

**Figure 6:**
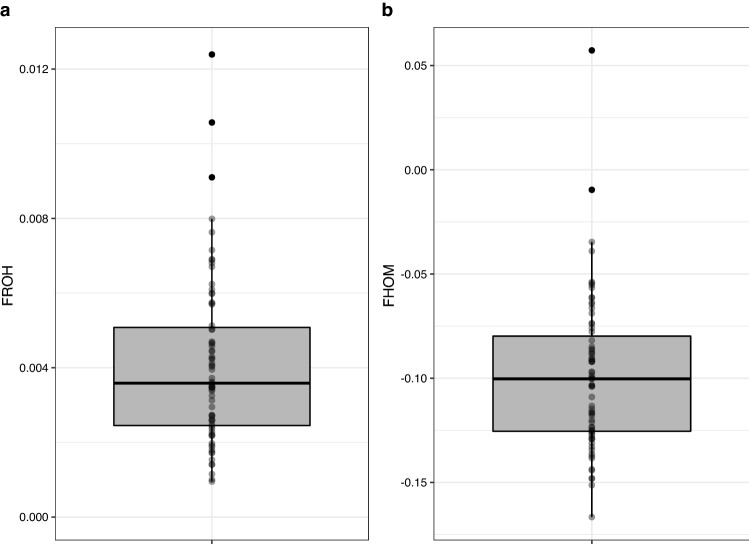
The distribution of *F* estimated from (**a**) runs of homozygosity (ROH) detection (F_ROH_) and (**b**) ROH coefficients (F_HOM_), across 71 African wild dogs (*Lycaon pictus*) from Kruger National Park (KNP), South Africa.

The estimated level of inbreeding among African wild dogs was based on ROH along the genome using a sliding window approach implemented in PLINK v1.90. A total of 7463 ROHs (> 500 kilobase pairs; kb) were detected across all genomes. The number of ROH segments per African wild dog ranged between 46 and 244 (Supplementary Table 1), with most individuals having between 81 and 127 ROH segments present in their genomes. The total length of the ROH segments (not contiguous) varied among African wild dogs (Fig. 3a), and the total ROH lengths were long in certain individuals (between ~ 34 mega base pairs; Mb and 191 Mb). However, the average lengths of most segments were below 1 Mb (Fig. 3b). Figure 4 shows the correlation between the number of ROH segments, and the total genomic length covered by ROH segments per individual, grouped by pack (Fig. 4a) and geographical location within KNP (Fig. 4b). The total genomic length (kb) covered by ROH per individual was approximately proportional to the total number of ROH per individual (Fig. 4b). There was no grouping of individuals based on pack identification or KNP location, although, individuals with the largest number of segments and total ROH length were from the southern and central parts of KNP (Fig. 4b). Two individuals from southern KNP and one from central KNP had ROH covering more than 150 Mb of their genomes. It appeared that most individuals had below 150 ROH segments and a total ROH length of less than 100 Mb. Figure 5a shows that the total ROH length consists of segments in the short length category (0.5–1.0 Mb) in all individuals. Figure 5b presents the average sum of the different ROH sizes across 24 African wild dog packs, revealing that most packs carried the highest total length of short ROHs (0.5–1.0 Mb). The inbreeding coefficients differed slightly; F_ROH_ was 0.0045 ± 0.0012 (Fig. 6a) on average across all individuals, and the mean F_HOM_ was -0.0963 ± 0.0350 (Fig. 6b).

## Discussion

In this study, we investigated key indicators of the genetic status of a stronghold African wild dog population in KNP, South Africa by using WGS data to explore levels of genomic diversity, relatedness, and inbreeding in 71 free-ranging African wild dogs. This sample size constituted 44% of the KNP population as determined by the 2017 annual population size of 163 ± 34^1^.

Using genome-wide SNP data, genomic diversity appeared to be low in the KNP African wild dog population, as expected. Several genetic studies conducted on African wild dog populations using different molecular genetic approaches have revealed a loss of genetic diversity. This has been especially evident in KNP populations, where Girman et al.^14^ reported lower mitochondrial variability in KNP individuals compared to other populations. Tensen et al.^19^ observed a loss in genetic diversity in selected genetic regions among African wild dog populations in the Greater Limpopo Transfrontier Conservation Area (reserves within South Africa, Mozambique, and Zimbabwe), which included a population from KNP. Specifically, allelic richness (diversity) and levels of heterozygosity in selected genetic regions have been lost over time due to inbreeding and genetic drift. Genetic structuring was most apparent in the KNP population as well as a Zimbabwean population due to a lack of gene flow caused by low dispersal rates^19^. Similar findings were observed in other African wild dog populations across Africa^10,20^, where low diversity, specifically at the adaptively important Major Histocompatibility Complex (MHC) loci (which affects disease susceptibility), was observed compared to other canids^46,47^. A follow-up study revealed a large decline in observed heterozygosity (compared to what was expected) at microsatellite and MHC loci in a population from KNP^20^. Additionally, this study also identified genetic signatures of a recent bottleneck in African wild dogs from South Africa (including KNP), Botswana, Tanzania, and Kenya, based on 10 microsatellite loci^20^.

A loss of genetic diversity is considered a common consequence of population bottlenecks^48–51^. Such demography may cause a population to be in mutation-drift disequilibrium and to undergo transient excess heterozygosity, which may last for a few generations. This bottleneck signature occurs due to the faster reduction in allelic diversity compared to heterozygosity^52–56^. The KNP African wild dog population showed significant heterozygosity excess (*P* << 0.0001), suggesting that this population is not at mutation–drift equilibrium, providing strong evidence for a population bottleneck. There is supportive evidence that southern (South Africa, Namibia, Botswana, Zimbabwe, Zambia) and eastern (Mozambique, Tanzania, Kenya, Ethiopia) African wild dog populations may have also suffered from bottlenecks^20,22,47^. These results suggest that the current KNP African wild dog population was derived from a small group of individuals (resulting from a bottleneck) with limited levels of genome variation.

The PCA revealed that the African wild dogs included in this study were genetically similar across all packs since there was no distinct pack clustering observed. There was a loose grouping of individuals based on their geographical locations (north, south, and central KNP), indicating subtle genetic structure, but this was not distinct enough to classify the population into subgroups. This finding, therefore, suggests minimal genetic drift between geographical locations due to the dispersal capacity of African wild dogs which facilitates gene flow between different packs and limits genetic isolation^57–60^.

As predicted, the relatedness analysis revealed that most of the KNP African wild dogs were distantly related (0–25% of their genomes were IBD) and approximately 15% were completely unrelated across the 24 packs. Very few first-order relationships (≥ 50% IBD) were identified by the relatedness analysis, although a portion of the pairs that were classified as second-degree relatives had relatively high relatedness measures (above 40% IBD), which may suggest that they classify as first-degree relatives since most of these pairs were pack mates. With the largest portion of the population being distantly related (low probability of sharing IBD alleles), it seems that the KNP African wild dogs avoided inbreeding within packs. Inbreeding avoidance has been proven to occur in African wild dogs^18^, forming part of their cooperative breeding system, and may explain the low relatedness. However, this avoidance mechanism can limit reproduction, exacerbating the problem of population decline. With the continuous reduction in population numbers due to environmental, anthropogenic factors, and subsequent genetic consequences, it is becoming increasingly important to include genetic data in conservation management to alleviate the loss of genetic diversity. This is especially crucial as evidence of restricted dispersal has been observed in certain areas^61^ and may occur more frequently with increased habitat fragmentation. Although dispersal increases gene flow between African wild dogs, it may become ineffective when the likelihood is low that the remaining African wild dog populations consist of unrelated individuals^14,15,62,63^. Therefore, genomic data are critical to ensure accurate measures of relatedness for conservation management. Incorporating genetic data into African wild dog management strategies has been successful in some studies^1,61^ and should be routinely employed in other conservation efforts. For example, investigating genetic structure among or across populations may provide insight into how efforts can be planned to increase connectivity and gene flow.

The identification of ROH in the genome was used to estimate the level of inbreeding in the KNP African wild dog population. The detection of ROH may also be informative of past demography, where long contiguous homozygous runs indicate recent inbreeding, and shorter segments suggest past consanguinity events in a population or a loss in genomic diversity due to a bottleneck or founder event^64–68^. More specifically, the length of ROH correlates to ancient and recent inbreeding due to recombination and can be used to infer the generations of inbreeding, indirectly revealing the demographic history of a population^49,69,70^. Long, contiguous ROH segments (above 10 Mb) are indicative of recent inbreeding, which could be traced to inbreeding events that occurred between common ancestors approximately five generations ago^71^. Short ROH segments reflect distant or ancient inbreeding, as recombination allows for the breakdown of segments over time^64,72,73^. Distant inbreeding can be classified as inbreeding events that occurred between 50 and 12.5 generations ago if the ROH lengths are between 1 and 8 Mb, respectively^70,71,74^. The ROH analysis provided evidence of distant inbreeding across this African wild dog cohort, with most of the average ROH lengths falling in the short ROH category (< 1 Mb). This indicates that inbreeding occurred between common ancestors approximately 50 generations ago, allowing for ROH decay to occur over a long period due to recombination. Although the number and total lengths of ROH segments varied among individual African wild dogs, there was a relatively large portion of African wild dogs that had less than 150 ROH segments with total lengths (not consecutive) of 100 Mb. Individuals with ROH exceeding 175 Mb were from the southern and central parts of KNP and the longest total ROH length in an individual was 191 Mb. The distributions of ROH segments using the total length of ROH values for different ROH track lengths revealed that among all individuals, the total lengths of short ROHs (between 0.5 and 1.0 Mb) were high. This was also the case when examining the total length over the five length categories per pack. This may suggest the presence of ancient relatedness or indicate a loss of genetic diversity resulting from a founder effect or a population bottleneck, which has also been observed in several wolf populations^49,52,55,66,69^. Both genomic inbreeding measures, based on genome-wide SNPs (F_HOM_ and F_ROH_), indicated low levels of inbreeding in this African wild dog cohort, corresponding to other studies that investigated inbreeding in this species^17,18^. The negative F_HOM_ value suggests that individuals had higher levels of heterozygosity than expected under HWE, which is a characteristic commonly observed in populations that have undergone a bottleneck, or that practice inbreeding avoidance behaviour^52,54,75–77^.

Although this is the largest cohort of African wild dogs to be included in a population genomic study using WGS, it does not eliminate the possibility of bias introduced when using genetic software tools designed specifically for large population sizes, such as estimates of heterozygosity and inbreeding coefficients^78,79^. For example, when heterozygosity estimates are based on SNPs only, the results are biased by sample size, where smaller populations produce larger estimates^56^. However, if a large number of markers are used, the issue of bias may be overcome^78^. Furthermore, the parameters used for the ROH analysis may have introduced biased results, as there are no guidelines for a robust and uniform ROH analysis for endangered wildlife species^80,81^. The LD pruning prior to ROH identification may have major effects on the ROH analysis^80^. A common goal with LD pruning for ROH detection is to exclude short, common segments that can be picked up as ROH, but have arisen from LD^82^. However, it has also been shown that LD pruning may prevent the identification of long ROH, which might have been the case in our study^80^. Additionally, the inconsistency among the conditions to define ROH limits the comparison between studies, since there is a lack of consensus across studies^83^.

## Conclusion

The results from this study suggested that the current genetic status of the KNP South African wild dog population is similar to other small, endangered species populations, given the low levels of neutral genomic diversity. There were very few closely related individuals and little evidence of recent inbreeding in this population. Additionally, the short lengths of ROH segments across the population were suggestive of ancient inbreeding which most likely occurred after a drastic population bottleneck, and together with inbreeding avoidance mechanisms, allowed for homozygous stretches to be broken down. Therefore, the long-term survival of this African wild dog population may not be immediately threatened by detrimental genetic factors characteristic of small populations, due to the absence of inbreeding depression^18^. However, the recovery of the population size of this species may not be achieved when inbreeding avoidance is practised, as it may cause population decline^18,63,75^. Consequently, the levels of genomic variation will likely remain low and may only be restored, at a slow pace, with the accumulation of mutations over several generations. With increasing human population growth and habitat fragmentation, there should be a focus on increasing the quality of habitat corridors to facilitate maximum dispersal (and gene flow). This is crucial given the small and fragmented remaining African wild dog populations. Inbreeding may increase over time when dispersal becomes restricted. Furthermore, knowledge of relatedness and inbreeding levels are important factors to consider when planning conservation strategies and may guide future African wild dog translocations and introductions intended to mimic natural dispersal patterns.

## Materials and methods

### Sample collection and DNA isolation

**Figure 7:**
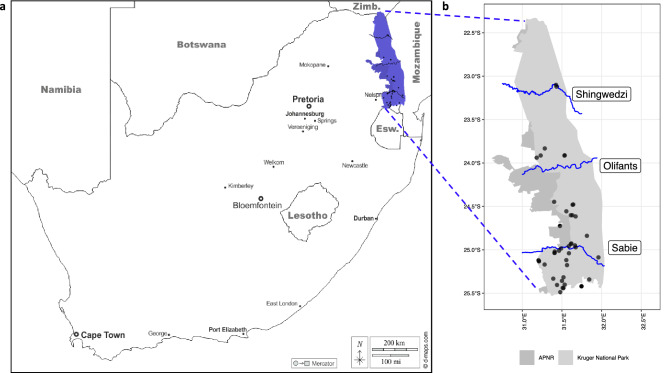
A map of South Africa (**a**), and the Kruger National Park (KNP) (21,353 km^2^) and Associated Private Nature Reserves (APNR) (**b**) with 71 African wild dog (*Lycaon pictus*) sampling locations (black dots). African wild dogs are widely distributed in KNP^85^, and the Sabie, Olifants, and Shingwedzi Rivers serve as natural geographical barriers and were used to classify the sampling locations into three groups. A single black dot may represent a sampling location for multiple African wild dogs (Supplementary Table 2). The figure of KNP was generated in R^97^ and the South African map was obtained from d-maps.com with permission and modified.

Samples were obtained from free-ranging African wild dogs (*n *= 71 individuals; 24 packs) from different regions of the KNP (Fig. 7; Supplementary Table 2) as part of a disease monitoring and vaccination project^84^. No inclusion or exclusion criteria were set during the sampling of African wild dogs. The sampling locations were classified into three sections: central, north, and south KNP. These sections were divided by three rivers within KNP, where sampling locations below the Sabie River were considered “south” (*n* = 37); locations above the Sabie and below the Olifants river were considered “central” (*n* = 21), and locations above the Olifants and below the Shingwedzi river were considered “north” (*n* = 13). African wild dogs were chemically immobilized according to South African National Parks Standard Operating Procedures for the Capture, Transportation and Maintenance in Holding Facilities of Wildlife (Buss P, personal communication). Due to logistical limitations of immobilizing an entire pack, not all individuals were sampled. Whole blood (2 ml) was collected in ethylenediaminetetraacetic acid (EDTA) tubes (Becton Dickinson, Franklin Lakes, New Jersey, USA) and stored at − 80 °C prior to DNA isolation. DNA was extracted from 200 μl of whole blood per sample using the Qiagen DNeasy Blood and Tissue Kit (Qiagen, Hilden, Germany) according to the manufacturer's instructions, with the following modifications: the volume of the elution buffer was reduced to 100 μl (instead of 200 μl), and the incubation period increased from 1 to 5 min after the elution buffer was added. Following extraction, the DNA concentration was measured spectrophotometrically using a NanoDrop 2000c (Thermo Fisher Scientific, Waltham, Massachusetts, USA). The integrity of the DNA samples was assessed by agarose gel electrophoresis (AGE), which was prepared at 0.8% using SeaKem® LE Agarose powder (Thermo Fisher Scientific) in sodium tetraborate buffer (SB buffer) and ethidium bromide (Merck, Kenilworth, New Jersey, USA). The gel was run at 120 V for 60 min. The DNA was stored at − 20 °C prior to WGS.

The protocol and procedures employed in this study were reviewed and approved by the Stellenbosch University Research Ethics Committee: Animal Care and Use (Ethics approval number: 6409). The methods in this study were performed in accordance to the Stellenbosch research ethics committee's guidelines and regulations for the Care and Use of Animal. Approval was also obtained from the South African Department of Agriculture, Land Reform and Rural Development (DALRRD) in terms of section 20 of the Animal Disease Act of 1984 (Act no. 35 of 1984) (Permit number: 12/11/1/7/2). Additionally, Biomaterial Transfer Agreements (BMTAs) were approved by the South African National Parks (SANParks) for all the African wild dog samples (Approval numbers: BMTA 002/18 and BMTA 003/19). The study is reported in accordance with the ARRIVE guidelines (https://arriveguidelines.org).

### Whole genome sequencing and quality control

All African wild dogs that had samples available were sent for WGS which included some members of the same pack. Library preparation and paired-end sequencing of 22 African wild dog DNA samples were undertaken at the Beijing Genomics Institute (Shenzhen, China). Whole genome sequences were generated as 100 base pair (bp) paired-end sequences at a depth of coverage of 30$\times$ per sample using the BGISEQ-500 sequencing platform. It is a combinatorial probe-anchor synthesis (cPAS)-based sequencing platform that combines DNA nanoball (DNB) nanoarrays with polymerase-based stepwise sequencing^45,86^. The library preparation and paired-end sequencing of the remaining 49 African wild dogs were performed at the South African Medical Research Council Genomics Centre, Cape Town, South Africa^87^. These samples were sequenced on the MGISEQ2000 platform at 15$\times$ coverage per sample and produced 150 bp paired-end sequences. One sample was sequenced on both sequencing platforms for comparison and the sample data from the MGISEQ2000 sequencing batch of this one sample was excluded from the statistical analysis because of a lower mean read depth (Supplementary Table 3). The read depth was calculated for each position using the -depth option in SAMtools^88^. The sequencing principle of the MGISEQ2000 platform depends on the DNB and cPAS technology but the reagents and software used have been refined and differed slightly from those used with the BGISEQ-500 platform^89^. FastQC v0.11.5^90^ was used for quality control of the sequence reads and to generate summary statistics for each sequence file. MultiQC v1.4^91^ was used to produce a single quality report of all the samples. To improve the quality of a sequence file, Trimmomatic v0.36^92^ was used to remove erroneous reads and trim low-quality bases toward the end of the reads.

### Read mapping and variant calling

All clean paired-end sequence reads were mapped to the high quality African wild dog reference genome (sis2-181106_HiC.fasta.gz) using BWA-MEM v0.7.17, and the -M (mark shorter split hits as secondary) parameter was implemented^21,93^. Picard v1.101^94^was used to add read groups to the sequence data and SAMtools^88^ was used to convert sequence alignment/map (SAM) files to binary alignment/map (BAM) files as well as merge BAM files for each individual. Reads that were not mapped to the reference genome were removed with SAMtools^88^. SAMtools was used to obtain mapping statistics of the sequences as well as to compute the depth and breadth of coverage of all the sequence reads. Variant calling was performed using bcftools mpileup v1.9^95^, and variants were removed if they had a base quality score (Q) of ≤ 30, if they deviated from HWE (*P* ≤ 1 $\times$ 10^–5^), and if they had a MAF of ≤ 0.05. Only biallelic SNPs were retained and a genotype call rate of 100% was used for this dataset. To reduce LD among SNPs, moderate LD pruning was conducted using PLINK v1.90 to remove SNPs within a 25 SNP window that had an *r*^2^ > 0.5 (--indep-pairwise 25 5 0.5). The stats command in bcftools and VCFtools v0.1.17^96^ was used to calculate the number of SNPs and the transition/transversion (Ts/Tv) ratio.

### Population genomic diversity and bottleneck

Genomic diversity indicators including expected (H_E_) and observed (H_O_) heterozygosity, were calculated using the --hardy flag implemented in PLINK v1.90^40,42^. Nucleotide diversity (π) was calculated for every 10,000 bases across the genome using the --window-pi option in VCFtools. To identify signals of a past bottleneck, the distributions of H_O_ and H_E_ were examined to determine if excess heterozygosity was present across the SNP dataset as well as a subset of SNPs that underwent stringent LD pruning (--indep-pairwise 200 20 0.2) in addition to MAF (≤ 0.05) and HWE (*P* ≤ 1 $\times$ 10^–5^) filtering. The difference between observed and expected heterozygosity (Δ = H_O_ − H_E_) was determined, where a positive difference (excess heterozygosity) was assumed to be indicative of a recent bottleneck^53^. The standardized difference statistical test developed by Cornuet and Luikart^53^ was used to test for significant excess heterozygosity. Specifically, a Test 2 (T_2_) statistic was calculated for each SNP (*L*), where ${\Delta }_{l}$= (H_O_−H_E_) for the $l$th locus and ${\sigma }_{l}$ was the standard deviation of H_O_ and H_E_ at the $l$th locus. The sum of these results was then multiplied by *L*^−0.5^ as follows^53^:

1 $$T_{2} = L^{ - 0.5} \mathop \sum \limits_{l = 1}^{L} \left\{ {\Delta_{l} \surd \sigma_{l} } \right\}$$

If excess heterozygosity is the alternative hypothesis, the null hypothesis (i.e., the population is in mutation-drift equilibrium) will be rejected at a 5% level if the T_2_ value is larger or equal to 1.645 (value from a table of normal distribution)^53^.

### Population structure

To examine the genetic structure among African wild dog packs as well as the structure among African wild dogs from different areas of KNP, a PCA was performed using PLINK v1.90 on SNPs (527,726) that were filtered based on overall quality, MAF, LD (moderate), and deviations from HWE. Additionally, the possibility of sequence bias (between samples sequenced on the BGISEQ-500 or the MGISEQ2000 platform) was also included in this analysis, since there was variability in the same sample on both platforms, regarding the number of reads produced and depth of coverage (Supplementary Table 3). PLINK v1.90 was used to compute the variance-standardized relationship matrix and R was used to plot the PC loadings^97^.

### Relatedness

A pairwise IBD analysis was performed using the original SNP data set (527,726 SNPs) to examine the first- and second-degree relationships among individuals as the proportion of the SNPs at which there were 0, 1 and 2 shared alleles IBD (IBD: probability that two alleles are descended from a single ancestor and are not identical by chance), represented as Z_0_, Z_1_, and Z_2_, respectively. Relatedness was calculated as the proportion IBD between individual pairs, as indicated by PLINK’s PI-HAT value: PI-HAT = P (IBD = 2) + 0.5 $\times$ P (IBD = 1). In this study, first-degree relatives were classified as having a PI-HAT value of at least 0.50, second-degree relatives of at least 0.25 and below 0.50, and distant relatives below 0.25 and above 0. The total number of pairs was calculated as follows^40,42^:

2 $$\frac{{k \times \left( {k - 1} \right)}}{2}$$

where *k* represented the number of samples included in the analysis.

### Inbreeding

Inbreeding history was estimated by analysing ROH within the genome using PLINK v1.90. To identify ROH segments within the filtered SNP data set (527,726 SNPs), the following conditions were applied:

- homozyg-snp 25—the minimum number of SNPs that an ROH was required to contain (25 SNPs);
- homozyg-kb 500—the length in kilobase pairs (kb) of the sliding window (500 kb);
- homozyg-density 25—the required minimum density to consider an ROH (1 SNP in 25 kb);
- homozyg-window-snp 25—the number of SNPs that the sliding window must contain (25 SNPs);
- homozyg-gap 1000—the length in kb between two SNPs to be considered in two different segments (1000 kb);
- homozyg-window-het (0–1)—the number of heterozygous SNPs allowed in a window (0 or 1);
- homozyg-window-missing 5—the number of missing calls allowed in a window (5 calls); and
- homozyg-window-threshold 0.05—the proportion of overlapping window that must be called homozygous to define a given SNP as within a “homozygous” segment (5%).

The ROH segments obtained were classified into various length categories or size bins (0.5–1.0; 1.0–1.5; 1.5–2.0, 2.0–2.5, and > 2.5 Mb) and the total length of ROH in each bin was calculated for each African wild dog. The total ROH length over the five length categories was also averaged per pack.

The genomic inbreeding coefficient was calculated based on genome-wide excess homozygosity due to inbreeding (F_HOM_) using PLINK v1.90^40–42^ as follows:

3 $$F_{HOM} = \frac{{H_{o } - H_{E} }}{{N - H_{E} }}$$

where H_O_ was the average observed number of homozygous markers, H_E_ was the expected number of homozygous markers under HWE, and N was the total number of markers. An additional measure of inbreeding was based on the proportion of the genome covered by ROH (F_ROH_) relative to the total length of the genome covered by SNPs computed using the detectRUNS package in R^97,98^ as follows:

4 $$F_{ROH} = \frac{{L_{ROH} }}{{L_{total} }}$$

where L_ROH_ was the total length of an individual’s ROH and L_total_ was the length of the genome covered with SNPs.

## Supplementary Information

Supplementary Information.

## Data Availability

The data for this study have been deposited in the European Nucleotide Archive (ENA) at EMBL-EBI under accession number PRJEB47265 (https://www.ebi.ac.uk/ena/browser/view/PRJEB47265).
