# Supplementary material for: African wild dogs (Lycaon pictus) from the Kruger National Park, South Africa are currently not inbred but have low genomic diversity
Source: Sci Rep. 2022 Sep 2;12:14979. doi: 10.1038/s41598-022-19025-7 (PMC9440078; doi:10.1038/s41598-022-19025-7)
Supplement: Supplementary file 1 — Supplementary Information. [file 41598_2022_19025_MOESM1_ESM.docx]

**African wild dogs (*Lycaon pictus*) from the Kruger National Park, South Africa, are not inbred but have low genomic diversity**

**Supplementary material**

Supplementary Figure 1: Distribution of heterozygosity across the original SNP dataset of 527,726 variants. **(a)** Density of H_O_ and H_E_ across 527,726 SNPs. **(b)** Scatter plot representing the correlation of H_O_ and H_E_ for each variant **(c)** Distribution of delta heterozygosity (Δ=H_O_−H_E_) across SNPs.

Supplementary Table 1: African wild dog samples included in this study, including age, sex and sampling location.

Supplementary Figure 2: Scree plot showing the percentage variance (eigenvalues) explained by 20 principal components (PC).

Supplementary Figure 3: Principal component analysis of 71 African wild dog samples (24 packs). Principal components 1 and 3; 2 and 3 are shown, with the proportion of variance accounted for by each indicated in parentheses. Each data point in the plot represents an individual, where each coloured shape corresponds to a pack (KNP_pack).

Supplementary Figure 4: Principal component analysis of 71 African wild dog samples. The first three principal components (PC 1, PC 2 and PC 3) are shown (a-c), with the proportion of variance accounted for by each indicated in parentheses. Each data point represents an individual, with the coloured shapes corresponding to the sequencing platform (Platform_seq).

Supplementary Table 1: Summary table of the runs of homozygosity (ROH) per African wild dog, including the number of ROH segments (NSEG), total length of ROH segments (KB) and average ROH length (KBAVG).

| **ID** | **NSEG** | **KB** | **KBAVG** |  |
| --- | --- | --- | --- | --- |
|  |  |  |  |  |

| LVS201605-011 | 102 | 70433.1 | 690.521 |
| --- | --- | --- | --- |
| LP-J-02 | 115 | 81815.2 | 711.437 |
| LP-K-02 | 110 | 86511.4 | 786.467 |
| LP-M-04 | 135 | 95503.1 | 707.43 |
| LP-N-01 | 134 | 102763 | 766.887 |
| LP-M-03 | 107 | 82978 | 775.496 |
| LP-M-05 | 92 | 67708.7 | 735.965 |
| LP-M-06 | 85 | 63225.2 | 743.825 |
| LP-E-04 | 143 | 107530 | 751.956 |
| LP-L-04 | 74 | 57379.1 | 775.393 |
| LP-N-03 | 99 | 74066 | 748.142 |
| LVS201607-004 | 98 | 77258.7 | 788.354 |
| LP-I-03 | 199 | 157871 | 793.324 |
| LP-L-01c | 128 | 91571.7 | 715.404 |
| LP-T-02 | 121 | 94654.8 | 782.271 |
| LP-O-03 | 69 | 52846.8 | 765.896 |
| LP-U-01 | 105 | 75468.2 | 718.744 |
| LP-P-01 | 70 | 51578.6 | 736.837 |
| LP-P-02 | 244 | 190793 | 781.939 |
| LP-Q-02 | 131 | 101441 | 774.356 |
| LP-Q-03 | 145 | 111715 | 770.451 |
| LP-K-04 | 122 | 86818.2 | 711.624 |
| LP-D-01 | 196 | 155611 | 793.933 |
| LP-K-05 | 96 | 70313.1 | 732.429 |
| LP-W-01 | 46 | 33534.1 | 729.001 |
| LP-E-05 | 88 | 62921.4 | 715.016 |
| LP-X-01 | 119 | 92791.9 | 779.764 |
| LP-X-03 | 63 | 52177.8 | 828.218 |
| LP-X-04 | 49 | 40754.4 | 831.723 |
| LP-R-01 | 165 | 142244 | 862.085 |
| LP-R-02 | 155 | 117504 | 758.089 |
| LP-R-03 | 97 | 67025.8 | 690.988 |
| LP-S-03 | 54 | 39109.7 | 724.254 |
| LP-B-04 | 155 | 121111 | 781.358 |
| LP-T-01 | 93 | 69304.6 | 745.211 |
| LP-T-03 | 136 | 106218 | 781.013 |
| LP-O-01 | 97 | 71591 | 738.051 |
| LP-U-02 | 132 | 103033 | 780.556 |
| LP-U-03 | 95 | 74566.8 | 784.914 |
| LP-U-04 | 114 | 91669.2 | 804.116 |
| LP-P-03 | 73 | 52918.3 | 724.908 |
| LP-Q-04 | 74 | 58679.6 | 792.967 |
| LP-V-01 | 133 | 103378 | 777.28 |
| LP-N-02b | 131 | 99939.5 | 762.897 |
| LP-D-02 | 125 | 94439.8 | 755.518 |
| LP-T-03 | 58 | 46942.5 | 809.354 |
| LP-A-01 | 98 | 71007.9 | 724.571 |
| LP-A-02 | 90 | 71260.7 | 791.786 |
| LP-A-03 | 114 | 86335.9 | 757.333 |
| LP-B-01 | 123 | 92387.2 | 751.116 |
| LP-B-02 | 63 | 49460.4 | 785.086 |
| LP-B-03 | 85 | 62080.1 | 730.354 |
| LP-I-01 | 102 | 75807.7 | 743.213 |
| LP-J-01 | 88 | 67021 | 761.602 |
| LP-D-03 | 66 | 50453.7 | 764.45 |
| LP-E-01b | 86 | 65260.8 | 758.846 |
| LP-K-01 | 79 | 62820.2 | 795.192 |
| LP-K-03 | 107 | 73991.5 | 691.51 |
| LP-L-02 | 84 | 64281.6 | 765.257 |
| LP-L-03 | 89 | 63673.3 | 715.431 |
| LP-M-01 | 64 | 49806.6 | 778.228 |
| LP-M-02 | 106 | 78711.1 | 742.558 |
| LP-E-02 | 96 | 69389.5 | 722.807 |
| LP-E-03 | 72 | 52949.6 | 735.411 |
| LP-F-03 | 48 | 40731 | 848.562 |
| LP-F-02 | 171 | 134767 | 788.109 |
| LP-G-02 | 82 | 61868.4 | 754.493 |
| LP-H-02 | 65 | 47895.9 | 736.86 |
| LP-I-02 | 81 | 56118.4 | 692.82 |
| LP-G-01 | 148 | 115026 | 777.203 |
| LP-H-01 | 84 | 70066.6 | 834.126 |

Supplementary Table 2: African wild dog samples included in this study, including pack, age, sex, sampling location, and the KNP section of the sampling locations.

| **ID** | **Pack** | **Age Class** | **Sex** | **Sampling Location** | **KNP Section** |
| --- | --- | --- | --- | --- | --- |
|  |  |  |  |  |  |
| LP-A-01 | A | Y.Adult | Male | Skukuza | South |
| LP-A-02 | A | Y.Adult | Male | Skukuza | South |
| LP-A-03 | A | Y.Adult | Female | Skukuza | South |
| LP-B-01 | B | Y.Adult | Female | Spanplekspruit | South |
| LP-B-02 | B | Adult | Female | Skukuza | South |
| LP-B-03 | B | Adult | Female | Skukuza | South |
| LP-B-04 | B | Adult | Female | Doispane Skukuza | South |
| LP-B-05 | B | Adult | Female | Kruger Gate | South |
| LP-D-01 | D | Adult | Male | Pretoriuskop | South |
| LP-D-02 | D | - | Female | Mestel Dam | South |
| LP-D-03 | D | Adult | Male | Mestel Dam | South |
| LP-E-01b | E | Adult | Female | Stolsnek | South |
| LP-E-02 | E | - | Female | Timfenene | South |
| LP-E-03 | E | - | Male | Timfenene | South |
| LP-E-04 | E | Adult | Female | Malelane | South |
| LP-E-05 | E | Adult | Male | Matjulu Windmill | South |
| LP-F-02 | F | Y.Adult | Female | Kingfisherspruit | Central |
| LP-F-03 | F | Y.Adult | Female | Kingfisherspruit | Central |
| LP-G-01 | G | Adult | Female | Pretoriuskop | South |
| LP-G-02 | G | Adult | Male | Pretoriuskop | South |
| LVS201607-004 | H | Adult | Female | Talamati | Central |
| LP-H-01 | H | Adult | Female | Lower Sabie | South |
| LP-H-02 | H | Adult | Male | Lower Sabie | South |
| LP-I-01 | I | Adult | Female | Skukuza | South |
| LP-I-02 | I | Adult | Female | Skukuza | South |
| LP-I-03 | I | Adult | Male | Western Boundry | Central |
| LP-J-01 | J | Adult | Male | Kwaggaspan | South |
| LP-J-02 | J | Adult | Male | Kwaggaspan | South |
| LP-K-01 | K | Y.Adult | Male | Crocodile bridge | South |
| LP-K-02 | K | Adult | Female | Crocodile bridge | South |
| LP-K-03 | K | Adult | Male | Crocodile bridge | South |
| LP-K-04 | K | Adult | Male | Malelane | South |
| LP-K-05 | K | Adult | Female | Malelane | South |
| LP-L-01c | L | Adult | Male | Mlambane River | South |
| LP-L-02 | L | Adult | Female | Malelane | South |
| LP-L-03 | L | Adult | Male | Malelane | South |
| LP-L-04 | L | Adult | Male | Bergendal | South |
| LP-M-01 | M | Adult | Male | Mudzanzeni | Central |
| LP-M-02 | M | Y.Adult | Female | Mudzanzeni | Central |
| LP-M-03 | M | Y.Adult | Female | Mudzanzeni | Central |
| LP-M-04 | M | Adult | Male | Mudzanzeni | Central |
| LP-M-05 | M | Adult | Male | Mudzanzeni | Central |
| LP-M-06 | M | O.Adult | Male | Mudzanzeni | Central |
| LP-N-01 | N | Adult | Female | Imbali | Central |
| LP-N-02b | N | Adult | Male | Imbali | Central |
| LP-N-03 | N | Adult | Male | Imbali | Central |
| LP-O-01 | O | Adult | Male | Tshokwane | Central |
| LP-O-03 | O | Adult | Female | Tshokwane | Central |
| LP-P-01 | P | Adult | Male | Skukuza | South |
| LP-P-02 | P | Adult | Female | Skukuza | South |
| LP-P-03 | P | Adult | Male | Skukuza | South |
| LP-Q-02 | Q | O. Adult | Female | Letaba-Phalaborwa Road | North |
| LP-Q-03 | Q | Adult | Male | Letaba-Phalaborwa Road | North |
| LP-Q-04 | Q | O. Adult | Female | Letaba-Phalaborwa Road | North |
| LP-R-01 | R | Juvenile | Female | Phalaborwa Gate | North |
| LP-R-02 | R | Adult | Male | Phalaborwa Gate | North |
| LP-R-03 | R | Adult | Male | Phalaborwa Gate | North |
| LP-S-03 | S | Y.Adult | Female | Skukuza | South |
| LP-T-01 | T | Y.Adult | Female | Phalaborwa | North |
| LP-T-02 | T | O.Adult | Male | Phalaborwa | North |
| LP-T-03 | T | Juvenile | Female | Phalaborwa | North |
| LP-U-01 | U | O.Adult | Male | Tamboti | Central |
| LP-U-02 | U | Adult | Male | Tamboti | Central |
| LP-U-03 | U | Adult | Female | Tamboti | Central |
| LP-U-04 | U | Y.Adult | Female | Tamboti | Central |
| LP-V-01 | V | Y.Adult | Female | Sabi Sands | Central |
| LP-W-01 | W | Adult | Female | Shingwedzi | North |
| LP-X-01 | X | Y.Adult | Male | Shingwedzi | North |
| LP-X-03 | X | Y.Adult | Female | Shingwedzi | North |
| LP-X-04 | X | Y.Adult | Female | Shingwedzi | North |
| LVS201605-011 | Y | Adult | Male | Tshokwane | Central |

O. Adult: old adult; Y. Adult: young adult

Wild dog age ranges:

• Juvenile: 0-1 year

• Young Adult: 1-2 years

• Adult: 3-5 years

• Old Adult: 6 years and above

Supplementary Table 3: Comparative analysis between BGISEQ-500 (BGI, China) and MGISEQ-2000 (SAMRC/BGI Genomics Centre, South Africa).

**Sample A China** **Sample A South Africa**

Instrument BGISEQ-500 MGISEQ2000

Number of reads produced 1,228,381,660 424,219,801

Mean read depth 50.99× 26.05×

Breadth of coverage 97.44% 97.41%

Mapping rate 98.52% 98.45%

Number of variants called 1,986,828 2,040,368

Number of indels called 666,097 679,948

BGI: Beijing Genomics Institute

SAMRC: South African Medical Research Council

Sample A: African wild dog LP-D-01
